# Identification and characterization of new structured RNA classes in plants

**DOI:** 10.1080/15476286.2025.2523696

**Published:** 2025-06-30

**Authors:** Maximilian Sack, Maren Reinhardt, Rica Burgardt, Philipp Berg, Julia Bauer, Andreas Wachter, Zasha Weinberg

**Affiliations:** a Interdisciplinary Centre for Bioinformatics and Bioinformatics Group, Department of Computer Science, Leipzig University, Leipzig, Germany; b Institute for Molecular Physiology (imP), University of Mainz, Mainz, Germany

**Keywords:** RNA secondary structure, alternative splicing, plants, covariation, *cis*-regulation, Non-coding RNA, snoRNA

## Abstract

Alternative splicing is a very important mechanism to diversify an organism’s transcriptome with minimal increases in genome size. It can modify the function of the finished protein or affect its regulation, e.g. induce nonsense-mediated decay (NMD) to degrade the transcript. Mechanisms that affect alternative splicing are therefore of great interest. It has been shown that splicing can be affected by RNA secondary structures within pre-mRNAs. These structured regions of RNA (strucRNA) would affect their transcript in *cis*, but only a few such cases are known in plants. In this study, we interrogate plant genomes for *cis*-regulatory strucRNAs. By applying a comparative-genomics-based approach to 130 plant genomes, we identified 16 strucRNA candidates. Five candidates likely regulate in *cis* using alternative splicing and NMD. Other predictions might not regulate alternative splicing, including four putative small nucleolar RNAs (snoRNAs). Of our five *cis*-regulatory strucRNAs that are implicated in alternative splicing control, two are now experimentally validated in follow-up studies. These results stand in contrast to the few previously validated examples. Although we were able to predict some strucRNAs, all motifs had generally modest levels of covariation, which is a pattern of mutations that indicates a conserved secondary structure. With few mutations, comparative-genomics-based approaches to find strucRNAs are less effective. Other approaches of finding regulatory RNAs in plants might thus be needed, and more available genomic or transcriptomic data might improve the quality and quantity of promising candidates.

## Introduction

Structured non-coding sections of RNA transcripts (strucRNAs) play an important role in gene regulation in pro- and eukaryotes [1–4]. Structures on pre-mRNAs may affect the expression of their parent genes in *cis* through alternative splicing (AS), which can for example change the function of the finished protein or induce nonsense-mediated decay (NMD) to degrade the transcript. Much research on strucRNAs has been conducted in bacteria and animals, while comparatively little has been done on plants. The few known examples of *cis*-regulatory strucRNAs that affect AS in plants are a thiamin pyrophosphate (TPP) riboswitch [5–7], an RNA thermosensor [8] and a plant 5S ribosomal RNA mimic (*P5SM*) [9].

These studies employed different methods to find their respective strucRNAs. The TPP riboswitch was identified in most clades of plants by sequence similarity to bacterial sequences by nucleotide BLAST. The RNA thermosensor was found through targeted computational and experimental structural analysis of the *HsfA2* gene from *Solanum lycopersicum* (tomato). The *P5SM* was found through computational prediction of sequence and structure conservation between non-coding regions of *Arabidopsis thaliana* (thale cress) and *Oryza sativa* (rice) related by protein sequence homology. Other studies have analysed plant genomes for conserved non-coding sequences (CNSs) [10–12], but only two [13,14] analysed these conserved regions for conserved secondary structures.

Structures that are conserved across multiple species allow for some sequence variation, as long as the base pairs that make up the structure persist, i.e. if a nucleotide of a pair mutates, the partner must mutate to conserve the structure. This feature of conserved structures is called ‘covariation’ and can serve as an indicator of a conserved RNA structure. A consequence of covariation is that it negatively affects the sequence conservation, requiring more sophisticated models to model and predict RNA structures [15–19]. Methods based on covariation have proven highly successful to discover novel strucRNAs in bacteria [20–27], vertebrates [28–31], fungi [32] and viruses [33].

With the success of previous studies using covariation to predict conserved RNA secondary structures in various phyla and the shortage of such studies on plants, we decided to pursue a comparative study of plants with an emphasis on covariation. We applied this approach in an analysis of all 130 species of plants from the RefSeq database [34] to find strucRNAs.

## Materials and methods

In the present study we focused on finding *cis*-regulatory strucRNAs in plants. The applied method is based on a previously established method [25] that was used to predict strucRNAs in bacteria. This method first clusters non-coding regions (NCRs) based on sequence similarity using nucleotide BLAST [35] homology and then uses CMfinder [36] to predict RNA structures in each cluster. As a first step for this study, this method was used with green algae genomes. This was done because the genomes of green algae are considerably smaller than those of land plants, thereby allowing the established method to be employed without significant modifications. Promising strucRNA candidates (called ‘motifs’) were evaluated alongside the results of our adapted method, as detailed below, and the results are presented together.

### Input set of non-coding regions

Due to the much larger genomes possessed by land plants, compared to green algae, we could not just cluster all NCRs based on sequence similarities, as this would have led to many false-positive matches and been quite computationally expensive. We instead opted to cluster proteins from *Arabidopsis thaliana* and *Oryza sativa* and use those to find homologous sequences in other species. We chose these species because they are the best-annotated plant species from two major groups of plants. We presume that most proteins of a given species should be homologs of a protein from *A. thaliana* or *O. sativa*. Proteins without homologs in these two species are likely present in a less diverse range of species; the lower diversity of any associated strucRNAs would likely result in less covariation, while even the covariation of widespread strucRNAs was often meagre (see Results).

We thus took all 48,265 protein sequences for *A. thaliana* from TAIR10.1 [37] and 42,580 protein sequences for *O. sativa* from IRGSP-1.0 [38]. These were clustered using CD-HIT [39] with an identity threshold of 0.8, resulting in 52,315 clusters of proteins. CD-HIT selects the longest sequence as a representative for each cluster and those were then searched against the protein sequences of all plant species in the RefSeq database version 204 [34]. This database contains 130 species from the Viridiplantae clade, commonly known as green plants, which includes green algae and land plants and those will henceforth be referred to as ‘our dataset’. The search was done using protein BLAST [35,40] with an E-value threshold of 10^−10^. This search could turn up hits to multiple proteins from the same species for one protein cluster, in which case we chose to take the five best scoring genes (including their protein isoforms) and discard the rest. This threshold of five was chosen because it includes almost 90% of all sequences, while avoiding large numbers of paralogs within clusters that might have a different function. If a cluster contained fewer than three genes, it was discarded. This resulted in 34,339 groups of similar proteins with at least three different genes each.

For each cluster of proteins, we then extracted all RNA sequences of the respective genes that are not always part of a coding transcript, based on RefSeq annotation. This set of sequences therefore contains all constitutively intronic areas. Additionally, areas of genes that are not constitutively exonic, i.e. cassette exons (CE) and alternative areas from intron retention, alternative 3’ and 5’ events, were also extracted.

We also analysed up to 1,000 nucleotides of sequence up- and downstream of coding regions of a gene to likely capture the entirety of both UTRs, as transcription boundaries are not reliably annotated in all species. This resulted in a total of 16 Gb (16 × 10^9^ bases) of NCRs near and inside genes to analyse, which equates to 15.3% of the whole dataset (104 Gb).

### RNA secondary structure prediction

RNA structural alignments were then inferred in each cluster of NCRs using CMfinder version 0.4.1.18 [25,36]. The resulting structural predictions were called ‘motifs’ and checked for overlaps to sequence matches of known RNA classes from Rfam version 14.10 [41,42]. The motifs were then scored using RNAphylo [43] and a combined score function from a previous study [25].

### Evaluation of the highest scoring motifs

The highest scoring motifs were evaluated manually through a process starting with additional homology searches using Infernal [44]. These were first performed on the NCRs from the motif’s original cluster, and then on all NCRs from our plant database. Additional sequences from these homology searches were added to the motifs and used to evaluate the predicted secondary structure, as well as the genomic context of the motifs. Motifs were rejected when searches revealed homologs that were incompatible with the predicted structure. Motifs that were borderline, e.g. lacked compelling covariation, were discarded when their homology searches revealed no additional sequences. We used CMfinder on motifs that had gained additional sequences to find alternative or better secondary structures.

We evaluated motifs based on evidence of covariation and surrounding genetic features, specifically the location of the individual sequences in or around genes. These genes were then investigated for homology and known (auto-)regulatory functions, especially if the motif is near alternative splice sites that are involved with NMD. This information was available for *A. thaliana* [45] and used to manually evaluate all motifs that contained sequences from *A. thaliana* and were nearby such splice sites. We classified motifs as promising candidates if they either (1) are found nearby alternative splice sites that are associated with NMD, while at least showing sequence conservation, or (2) exhibit compelling covariation, in our subjective judgement. We evaluated a motif’s covariation as compelling if the sequences show homology, the covarying columns contain Watson-Crick base pairs and the alignment does not contain too many insertions. Sequences should be located in related introns of homologous genes or they would undermine the hypothesis for a conserved *cis*-regulatory function. Non-homologous sequences may fit the same secondary structure, but a lack of sequence conservation undermines the validity of the observed covariation. Sometimes CMfinder can misalign even homologous sequences, for example, by inserting gaps in a way that appears to improve covariation, but compromises sequence conservation. Due to this, the alignments of the highest scoring motifs had to be evaluated manually.

After this process, motifs were again compared to Rfam and representative sequences were searched in RNAcentral [46]. Studies about genes in which these motifs occur were also searched to ensure that no regulatory RNAs were already known and to aid in formulating hypotheses about the strucRNAs’ functions.

Finally, the significance of the predicted secondary structures was evaluated using R-scape [47] using a modified procedure. R-scape first creates a phylogenetic tree that explains all sequences from an alignment through as few mutations as possible. This tree is used to generate random sequences that follow the sequence of mutations in the original alignment. These random sequences are then used to gauge how likely it is to gain the covariation observed in the original alignment by chance. If the putative covarying base pairs appear through random chance, it argues against the biological importance of the predicted structure. On the other hand, if the covariation from the original alignment is unlikely to form by chance, evidence arises in support of the proposed structure. This analysis requires the sequences to be correctly aligned so that mutations can be identified and a bias in the original alignment will be reflected in the calculated Evalues.

The predicted alignments are biased towards fitting the predicted secondary structure, because of the use of CMfinder and Infernal. To remove this bias, a homology search was conducted on our RefSeq plant database without any base pairs (i.e. ignoring any secondary structure), using a single representative sequence for each motif. The resulting alignments had their original structure reapplied, without modifying the alignment, and were evaluated using R-scape. The E-values per base pair were aggregated for each stem by R-scape using the Lancaster method [48]. If a motif had multiple stems, the lowest of the aggregated E-values was used to evaluate the significance of the motif, since the presence of at least one significant stem implies the existence of some secondary structure. We considered predicted secondary structures to be significant based on R-scape if this minimum aggregated E-value was less than 0.05. This method was inspired by a previous approach using R-scape [32]. RNA structures were drawn using R2R [49] and Inkscape (https://inkscape.org/) and covariation was annotated based on R-scape.

### Plant material and growth conditions

All *A. thaliana* lines were in the Col-0 background. The NMD mutants *lba1* [50], *upf3-1* [51] and the double mutant *lba1 upf3–1* [45] were previously described. The double mutant is seedling-lethal and was generated by self-pollination of *lba1*^−/−^ *upf3–1*^+/-^ or *lba1*^+/-^ *upf3–1*^−/−^ plants. Only the 25% of their progeny that showed early developmental arrest and were therefore homozygous for both alleles were used for experiments. *A. thaliana* was grown in sterile culture, seeds were surface-sterilized with 3.75% NaOCl and 0.01% Triton X-100 and sown onto half-strength Murashige and Skoog (MS) medium including vitamins containing 2% sucrose and 0.8% plant agar (Duchefa). Plates were kept at 4°C in darkness for 1–2 days to synchronize germination before transferring them to a climate chamber. *Physcomitrella patens* (stock provided by International Moss Stock Center, Freiburg) was also kept in sterile culture on half-strength MS medium including vitamins with 0.8% plant agar, but without sucrose. *Arabidopsis lyrata* was grown on soil and rosette leaves were used for analysis. *Brassica rapa* (seeds provided by INRAE Biological Resource Center BrACySol, Rennes), *Glycine max* and *Zea mays* (seeds provided by Botanical Garden, University of Mainz), and *S. lycopersicum* were also grown on soil and harvested at the seedling stage. Growth conditions were as follows: 16 h light/22°C, 8 h darkness/20 °C, ~100 µE white light, 60% relative humidity. In case of *Populus trichocarpa*, leaf material was provided by the Botanical Garden, University of Mainz.

### Alternative splicing analyses

Isolation of total RNA was performed from ~100 mg plant tissue using the Universal RNA Purification Kit (Roboklon), including an on-column DNase treatment according to the manufacturer’s instructions. Reverse transcription was performed with a dT20 primer and SuperScript II Reverse Transcriptase (Invitrogen) or, in case of the NMD mutant data, AMV Reverse Transcriptase Native (Roboklon). Splicing variants were co-amplified by standard PCR procedures using the primers indicated in Supplementary Table S2. PCR products were separated on an agarose gel and visualized by ethidium bromide staining. Quantification was performed with an Agilent Bioanalyzer 2100 according to the DNA1000 protocol. Splicing variants were confirmed by cloning into pGEM-T (Promega) and Sanger sequencing.

## Results

We focused on finding *cis*-regulatory strucRNAs and therefore interrogated homologous NCRs for conserved secondary structure. We used proteins from *A. thaliana* and *O. sativa* to find homologous genes and extract their NCRs.

Clustering of *A. thaliana* and *O. sativa* proteins using CDHit [39] and finding homologous proteins in RefSeq [34] using protein BLAST [35] resulted in 34,339 clusters of proteins with at least three genes each. NCRs within and adjacent to genes were then extracted for the genes in each cluster. To search for conserved strucRNAs within those clusters, we used a previous system [25]. Alignments and secondary structures were predicted using CMfinder version 0.4.1.18 [25,36] for sequences in each cluster, resulting in 490,000 total structural predictions. These were termed ‘motifs’ and the ~ 6,800 highest-scoring motifs were manually evaluated.

Our highest scoring motifs contained promising predictions that overlapped known RNA classes, including the previously described *P5SM* [9] and TPP riboswitch [5,7]. This suggests that the method can detect *cis*-regulatory plant RNAs and assign them high scores. Most of the manually evaluated motifs were not further pursued due to a lack of compelling covariation and sequence conservation. The motifs that showed sequence conservation and signs of covariation were analysed further.

### Strongest candidates for strucRNAs

We present the most promising motifs (see Materials and methods) predicted by our methods in the following sections. For all motifs, we provide a list with summary data (Supplementary Table S1), machine-readable alignments (Supplementary File S4) and depictions of structural features (Supplementary File S5). Since the biological functions of most motifs remain unknown, we named them after associated genes or clades of host species. The motif summary data include statistical significance of the motif’s covariation based on the R-scape software (see Materials and methods).

Additionally, we had R-scape evaluate all possible base pairs in each motif in an effort to find additional base pairs beyond those in our predicted secondary structures. (Note that this is distinct from our other usage of R-scape to validate our proposed structures by only considering pairs in that structure and by aggregating covariation within a helix.) However, in our application of R-scape to all possible pairs, pairs that it calculated as showing significant covariation were already part of the secondary structures that we predicted (data not shown). Only 4 of our 16 motifs in plants contain significantly covarying base pairs with an average of 0.625 pairs per motif (Supplementary Table S1). We applied this method to a previous study in bacteria on RNA structural motifs [52], and found that 22 of their 23 predicted motifs contain between 1 and 21 significantly covarying base pairs, with an average of 6.7.

Of the 16 motifs that we present (Table 1), 14 appear in consistent locations within either UTRs or introns in homologous genes. These motifs could be *cis-*regulatory, but additional evidence of *cis*-regulation was only found for five of them, while four of them show features of snoRNAs.

**Table 1.** Summary of the most promising motifs.

Motif name	Clade	Function	Genes	Position	NMD	E-value
*DEAD*	*Tracheophyta, Bryophyta*	*cis*-reg	*RH14 (DRH1), RH46*	Intron/Alt3’	Alt3’	*0.0044
*45ABC*	*Magnoliophyta*	*cis*-reg	*RBP45A, RBP45B, RBP45C*	Intron/CE	CE	0.9
*GRP7&8*	*Pentapetalae*	*cis*-reg	*GRP7, GRP8*	Intron/Alt5’	Alt5’	*0.036
*PTB2*	*Pentapetalae*	*cis*-reg	*PTB2*	Intron/CE	CE	0.87
*BPM1&2*	*Rosids*	*cis*-reg	*BPM1, BMP2*	Intron/CE	CE	0.23
*MAF1*	*Magnoliophyta*	–	*MAF1*	Intron/CE	–	*3.7×10^−5^
*UbiE2*	*Pentapetalae*	–	*UEV1D-4 ubiquitin E2*	Exon, 5’UTR	–	*0.026
*Kre33*	*Mamiellales*	–	*Kre33*	Intron	–	*3.0×10^−7^
*STK38*	*Magnoliophyta*	–	*STK38*	Exon, 3’UTR	–	0.54
*MicMet*	*Micromonas*	–	*S*-adenosylmethionine synthase, cystathionine gamma-synthase	3’UTR	–	*6.4×10^−11^
*PRP38-HACA*	*Magnoliophyta*	snoRNA	*PRP38*	Intron	–	*5.7×10^−4^
*MPCRibo-HACA*	*Magnoliophyta*	snoRNA	Mitochondrial phosphate carrier protein 3; 40S & 60S ribosomal proteins	Intron	–	*0.0016
*Mamiellales-CD*	*Mamiellales*	snoRNA	uncharacterised	Intron	–	*0.001
*EIF3G1&2-HACA*	*Magnoliophyta*	snoRNA	*EIF3G1, EIF3G2*	Intron	–	*3.4×10^−6^
*Viridi-trans*	*Viridiplantae*	*trans*	–	–	–	*1.6×10^−6^
*Brassica-trans*	*Brassicaceae*	*trans*	–	–	–	*0.0036

Motifs are listed in the same order as in the manuscript text. Function: Hypothesis about the motif’s function (,,*cis*-reg“ = ,,*cis*-regulatory“, a dash is used for motifs that do not permit a prediction with reasonable confidence); Genes: Genes in which sequences from the motif are found, if consistent, otherwise a dash; NMD: If motif is nearby to an NMD-associated splice site, what type of splice event is it associated with; E-value: E-value calculated by R-scape for an alignment created through structureless homology searches (see Materials and methods), where an asterisk indicates E-values better than 0.05. A more detailed version of this table is available as Supplementary Table 1.

### Cis-regulatory strucRNAs associated with NMD

Five of the *cis*-regulatory motifs have alternative splice sites in their vicinities that are annotated in *A. thaliana* and lead to NMD of the respective splicing variant [45]. Thus, we hypothesize that these strucRNAs influence the expression of their parent genes by affecting AS and generating transcripts targeted by NMD. Precedents for this broad mechanism in plants are the TPP riboswitch [5,7] and the *P5SM* [9]. Four predicted *cis*-regulatory motifs in the present study are found in genes whose products directly interact with RNA. In these cases, we hypothesize that the finished protein might interact with the predicted structure on the pre-mRNA. This interaction could result in a conformational change, allow recruitment of other factors or prevent recognition of the splice site, and ultimately initiate NMD. These hypotheses will need to be analysed experimentally. The motifs yield clear models of their biological function and exhibit covariation that is qualitatively compelling (see Methods), even if not entirely convincing. However, most do not exhibit statistically significant covariation, according to the R-scape-based method. One motif lacking statistically significant covariation, the *45ABC* motif, has had its predicted secondary structure partially validated by structural mutant experiments (Reinhardt et al., in preparation), supporting the qualitative judgement of its covariation.

### DEAD motif

**Figure 1.**
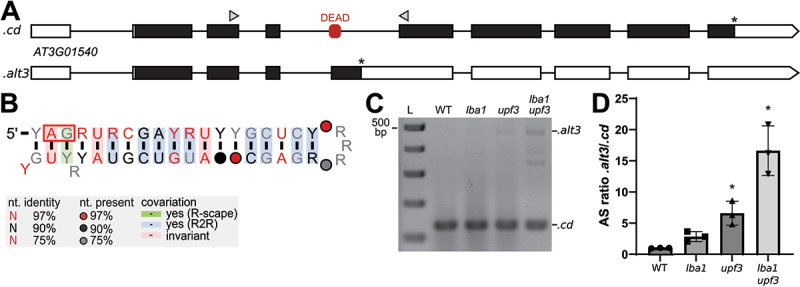
Alternative splicing in *AT3G01540* (*DRH1*) in the region surrounding the *DEAD* motif. (A) Gene models of splicing variants .*cd* and .*alt3* with exons (boxes) and introns (lines). Boxes correspond to UTRs (white) and coding sequences (black). The rounded red rectangle indicates the *DEAD* motif. Asterisks indicate positions of translational stop codons, grey arrowheads show binding sites of primers for co-amplification PCRs in (C) & (D). (B) Secondary structure predicted for the *DEAD* motif. The multiple sequence alignment that this secondary structure is based on, along with all other alignments presented in this study, can be found in Supplementary File 4. Red box indicates alternative 3’ splice site. Nucleotide color and symbol indicates level of nucleotide conservation. Coloration of the base pairs indicates level of covariation observed. Green: covariation based on R-scape’s robust statistics, blue: tentative covariation based on R2R, red: invariant, no coloration: unconserved. (C) RT-PCR products of major splicing variants detected from *A. thaliana* wild type (WT) and NMD-impaired mutant seedlings (see Materials and methods). L, size marker in 100 bp increments from 100 to 500 bp. (D) Ratio quantification of splicing variants shown in (C) via Bioanalyzer. Data are normalized to WT and represent mean values +SD, with three replicates for each genotype, marked with symbols. Statistical test: one-sample t-test against theoretical mean of 1.0, *P < 0.05.

One of the most promising motifs occurs in DEAD-box RNA-helicase genes in 115 species from the *Embryophyta*, also called land plants. We termed the motif the *DEAD* motif since it occurs in two genes from *A. thaliana*: *DEAD-box RNA-helicase 1* (*DRH1*, also called *RH14*, with locus tag *AT3G01540*) and a DEAD-box RNA-helicase family gene (*RH46*, *AT5G14610*). The motif overlaps an alternative 3’ splice site (Figure 1(a)), based on AS data [45]. The predicted secondary structure is a hairpin loop (Figure 1(b)) and it has previously been reported by Burgess and Freeling [14] for sequences from *A. thaliana* and *O. sativa*. The sequences of this motif in *A. thaliana* were also reported by Akkuratov [13] as evolutionarily conserved.

We show that homologs of this motif are found in DEAD-box RNA-helicase genes from all species of the *Tracheophyta* and *Bryophyta* clades that are in our dataset. Based on subjective and quantitative analyses, we conclude that this predicted secondary structure is supported by conservation and covariation of nucleotides across those species. To evaluate the covariation objectively, we analysed the resulting alignment using a carefully designed process utilizing R-scape. R-scape calculated an E-value of 4.39 × 10^−3^ for the motif’s single stem, which led us to reject the null hypothesis that the observed covariation occurred by chance.

In *A. thaliana DRH1*, the motif is present in the 4^th^ intron of its parent gene, overlapping an alternative 3’ splice site (Figure 1(a,b) red box). We hypothesized that the predicted secondary structure influences splicing to regulate expression of the gene. To investigate this hypothesis, we amplified *A. thaliana* cDNAs using RT-PCR with primers encompassing the motif-containing region, and detected the major splicing variant *AT3G01540.cd* and the less abundant variant *AT3G01540.alt3* (Figure 1(a,c)).

Cloning and sequencing of the RT-PCR products revealed that the two splicing variants result from an alternative 3’ splice site choice in the motif-containing intron (Figure 1(a)). Co-amplification of the two AS variants in seedling samples derived from WT and the NMD-impaired mutants *lba1*, *upf3*, and the double mutant *lba1 upf3* [45] showed a relative accumulation of *AT3G01540.alt3* upon NMD impairment (Figure 1(d)). Accordingly, we conclude that the AS variant *AT3G01540.alt3* is likely targeted by NMD, which is in line with the presence of a long 3’ UTR resulting from a premature termination codon (PTC), a classical feature of NMD targets. Regulated formation of varying proportions of the splicing variants *AT3G01540.cd* and .*alt3* would thus allow quantitative gene control, and the presence of the *DEAD* motif in the alternatively spliced region suggests a function of this motif in controlling the AS decision.

After confirming AS for *A. thaliana*, we evaluated the conservation of the splice site and its association with AS bioinformatically and experimentally for other species in which the motif is present. Bioinformatically, we observe strong conservation of the ‘AG’ dinucleotide in the alignment that corresponds to a 3’ splice site in *A. thaliana*. The ‘A’ (adenine) is conserved in all sequences and the ‘G’ (guanine) is conserved in 94% (319/339) of sequences. Further inspection of the sequences that do not conserve the ‘G’ showed that most (16/20) are found in *Ipomoea triloba* and *Ipomoea nil*. In these sequences, no genomic features are annotated nearby, with one exception in which a predicted *DEAD* motif is found inside an uncharacterized gene. That gene’s protein does not match DEAD-box RNA helicases, according to protein BLAST. In all of those sequences where ‘G’ is replaced by ‘C’ (cytosine), ‘A’ or ‘U’ (uracil), an accompanying covariation of their original binding partner (‘C’) in the *DEAD* motif to a ‘G’, ‘U’ or ‘A’, respectively, conserves the predicted secondary structure. The deviant sequences include two sequences from unplaced genomic scaffolds from *Nicotiana tabacum* with no genes annotated nearby, and for those, the ‘G’ was changed to an ‘A’ and their binding partners to a ‘U’, thereby conserving the predicted secondary structure. The two remaining sequences lacking the ‘G’ from the splice site marker are found in *Capsicum annuum* and *Rosa chinensis* inside annotated DEAD-box RNA helicase genes. For these two sequences, the ‘G’ was changed, but its binding partner did not change to conserve binding.

For all of these sequences that do not conserve the splice site, there are two sequences each from the same species that fit the motif very well, including the splice site, and are found in DEAD-box RNA helicase genes. The sequences lacking the AG could therefore be in paralogs that are regulated differently, gained different functions or are pseudogenes; in these cases, regulation through the motif might no longer be relevant. Although these sequences appear inconsistent with our model that the *DEAD* motif regulates its gene based on AS at an internal 3’ splice site, their E-values in covariance model searches met our threshold of 0.05, and were therefore retained in our alignment. If our hypothesis of regulation via alternative splice sites is correct, despite these exceptions, all or most genes with the motif should produce different transcript variants due to an alternative 3’ splice site. We therefore investigated AS of this gene in other plant species that contain the motif.

**Figure 2.**
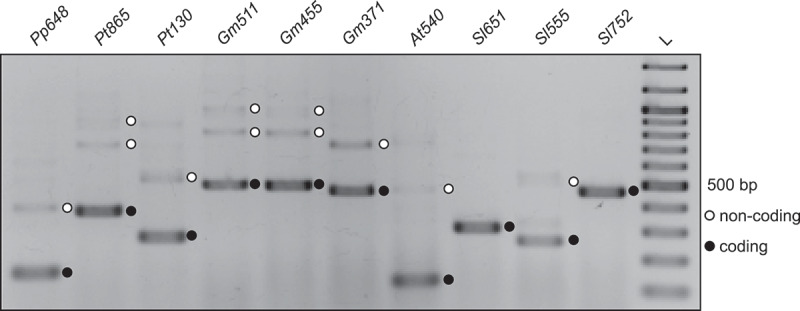
Alternative splicing of *DEAD* homologs in various species. Species were selected to cover different plant lineages and based on availability of genetic material. RT-PCR products were separated on an agarose gel and visualized by ethidium bromide staining. The lower, most dominant band corresponds to splice variant .*cd* in each lane. The species and loci are: *Pp648*: *Physcomitrella patens*, locus tag *LOC112288648; Pt865: Populus trichocarpa, LOC7487865; Pt130: P. trichocarpa, LOC7468130; Gm511: Glycine max, LOC100786511; Gm455: G. max, LOC100816455; Gm371: G. max, LOC100806371; At540: Arabidopsis thaliana, AT3G01540; Sl651: Solanum lycopersicum, LOC101248651; Sl555: S. lycopersicum, LOC101266555; Sl752: S. lycopersicum, LOC101254752*. For *Sl651* and *Sl752*, only the coding variant could be confirmed in the analysed samples. L, size marker in 100 bp increments from 100 to 1000 bp, plus 1200 and 1500 bp bands. Gene models for all transcripts are depicted in Supplementary Figure 1 in Supplementary File 1 and sequences are in Supplementary File 2.

Using RT-PCR and sequencing, we show that the usage of the alternative 3’ splice site within the *DEAD* motif is conserved (Figure 2). Such a conservation of AS events over multiple species is quite rare [53,54] and could indicate that the function of the motif and its connection to AS is important. In all cases where there is AS near the motif, the coding variant is the most abundant one and the alternative variants have premature termination codons (Supplementary Figure S1 in Supplementary File S1). This suggests that regulation of these loci via NMD through AS is also conserved across the sampled species, and by extension across all or most loci that the motif is found in.

Furthermore, since DRH1 is an RNA helicase and the *DEAD* motif contains a predicted large helix, targeting of the pre-mRNA by the finished protein seems plausible. Burgess and Freeling [14] hypothesized that the regulation of translation of the RNA might involve unwinding of this secondary structure by the finished protein. With the information on AS transcripts, we can propose an alternative hypothesis: elevated helicase activity would unwind the *DEAD* motif’s helix, thereby making the alternative splice site available and preferentially forming the unproductive variant .*alt3*, leading to diminished gene expression. Further analysis of this motif and its autoregulation will be published in a separate study (Burgardt et al., in preparation)

### 45ABC motif

**Figure 3.**
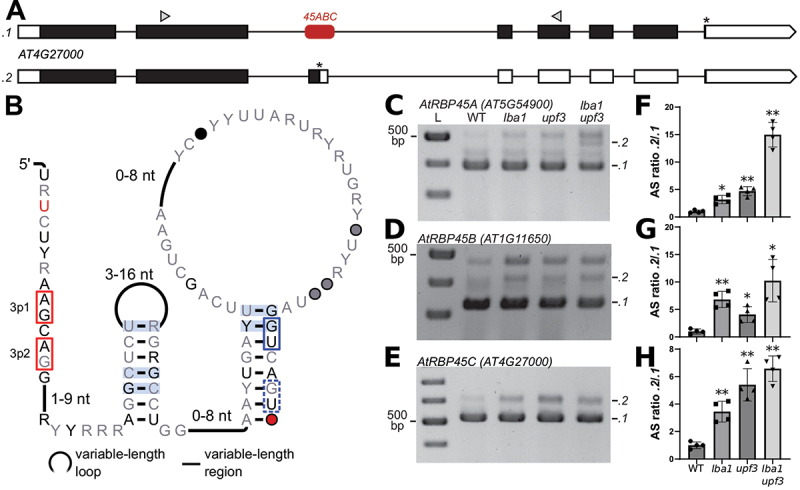
Alternative splicing associated with the *45ABC* motif. (A) Gene models of splicing variants .*1* and .*2* derived from *AtRBP45C* (*AT4G27000*). Rounded red rectangle indicates the *45ABC* motif. Gene model structure described in legend to Fig. 1A. (B) Predicted secondary structure model of the *45ABC* motif; nucleotide annotation as described in Fig. 1. Alternative 3’ and 5’ splice sites used for cassette exon inclusion are indicated by red and blue boxes, respectively. The dashed blue box indicates a conserved “GU” dinucleotide that is not annotated as an AS site. (C,D,E) RT-PCR products in *A. thaliana* WT and NMD mutant seedlings for *AtRBP45A* (*AT5G54900*), *AtRBP45B* (*AT1G11650*) and *AtRBP45C* (*AT4G27000*). Gene-models for *AtRBP45A&B* are depicted in Supplementary File 1. L, size marker in 100 bp increments; 300, 400, 500 bp for C&BD, 400, 500, 600, 700 bp for E. (F,G,H) Bioanalyzer quantification of ratios of major splicing variants from the motif-containing *RBP45* genes in *A. thaliana* WT and NMD mutant seedlings. Data are normalized to WT and represent mean values +SD, with four replicates for each genotype, marked with symbols. Statistical test: one-sample t-test against theoretical mean of 1.0, *P <0.05, **P < 0.01.

Another promising motif was found in paralogous RNA-binding protein genes called *RBP45A*, *B* and *C*, and therefore termed the *45ABC* motif. It is positioned in an intron and overlaps a CE (Figure 3(a)). Burgess and Freeling [14] previously predicted sequences in *A. thaliana* and *O. sativa* that overlap this motif as a conserved non-coding sequence. We predict the secondary structure as two hairpin loops with the splice sites of the CE being at the start and end of the motif (Figure 3(b), red and blue boxes).

We expand upon previously published data with sequences from almost all 113 available species of the *Magnoliophyta*, also called flowering plants. We did not find the motif in *Lupinus angustifolius*, which is a *Magnoliophyta* and is available in our dataset based on RefSeq, resulting in 112 species for this motif. We found compelling covariation to support our predicted secondary structure. This covariation, however, is only subjectively encouraging and more promising than most other motifs we analysed, whereas R-scape calculates an E-value of 0.9 in our procedure, which is not significant.

We analysed alternative splice sites in this motif in a similar manner to the procedure conducted for the *DEAD* motif. In *A. thaliana*, the motif was identified in three genes: *AtRBP45A* (with locus tag *AT5G54900*), *AtRBP45B* (*AT1G11650*) and *AtRBP45C* (*AT4G27000*), with *AtRBP45B* not being predicted previously [14]. For *AtRBP45B* and *AtRBP45C*, two alternative 3’ splice sites are annotated immediately next to one another (3p1 and 3p2) (Figure 3(b), red boxes), forming a NAGNAG site, where N represents any nucleotide and splicing at either site results in the same reading frame. For *AtRBP45A*, only the second alternative 3’ splice (3p2) site is annotated. We analysed RT-PCR products of *AtRBP45* transcripts and identified two major AS variants resulting from cassette exon inclusion or skipping for all three genes (Figure 3(c–e)). Skipping of the cassette exon generates a splicing variant that encodes the full-length protein, while inclusion of this exon, using either alternative 3’ splice site, introduces a PTC and a long 3’ UTR, typical NMD target features. In all three examples, the alternative 3’ and 5’ splice sites used for the inclusion of the cassette exon lie within the *45ABC* motif (e.g. see Figure 3(b)). Comparing the AS ratios between samples derived from WT and NMD-impaired mutant seedlings revealed a relative accumulation of the .*2* variants upon NMD impairment (Figure 3(c–h)). Accordingly, inclusion of the cassette exon triggers NMD of the corresponding splicing variants. These results are in line with previous splicing analyses of *AtRBP45B* (*AT1G11650*) in NMD mutants [45].

We investigated the nucleotide conservation of dinucleotides in the *45ABC* motif that correspond to annotated NMD-associated alternative splice sites in *A. thaliana*. Annotations in RefSeq are more common for the second 3’ splice site (3p2) than the first one, and only annotate the first one (3p1) if the second one is also annotated. The following analysis suggests that the precise splice sites might not be as important as the presence of a PTC upon exon inclusion. Even though splice site 3p2 is more frequently annotated, it is less conserved in our alignment: 4.2% (19/448) of sequences do not conserve the ‘A’ and 6% (27/448) do not conserve the ‘G’ of the ‘AG’ splice site marker dinucleotide. For 3p1, the ‘A’ is absent from only 0.7% (3/448) sequences and the ‘G’ is absent from 4.7% (21/448) sequences.

One reason for apparently low conservation of 3p2 are the 16 sequences in the alignment from *Arachis hypogaea*, *Arachis ipaensis* and *Arachis duranensis* that are completely missing 3p2 as well as an additional nucleotide (‘AGG’). The absence of these three nucleotides preserves the reading frame and thus the PTC. However, there are no genes annotated around these sequences and usage of BLAST to look for protein-coding genes only found genes encoding proteins of uncharacterized functions. We also found 13 sequences in *Papaver somniferum* that have no genes annotated in their vicinity, with a ‘AU’ dinucleotide instead of the ‘AG’ for 3p1 and/or 3p2. In terms of 5’ splice sites, the sequence consensus of the motif shows two possible 5’ splice sites at the 3’ end of the motif (Figure 3(b), blue boxes). Only the first one of the two is annotated as an alternative splice site in *A. thaliana*, and the second ‘GU’ is likely part of the 5’ splice site consensus ‘GUAAGU’ [55] and not a separate splice site. The 5’ splice site is not conserved in 14 sequences from *Gossypium hirsutum* and *Gossypium raimondii* that are inside uncharacterized or hypothetical genes, whose proteins do not match any RBP45 proteins. All of them have an ‘AU’ instead of a ‘GU’ dinucleotide for both possible 5’ splice sites. For each sequence in the motif that does not conserve the splice site markers, there is at least one sequence from the same species that is in an *RBP45* gene and conserves the splice site markers. As with the *DEAD* motif, one could argue to remove those sequences from the alignment due to missing gene homology, but we decided to keep them due to their sequence and structural similarity to the motif.

**Figure 4.**
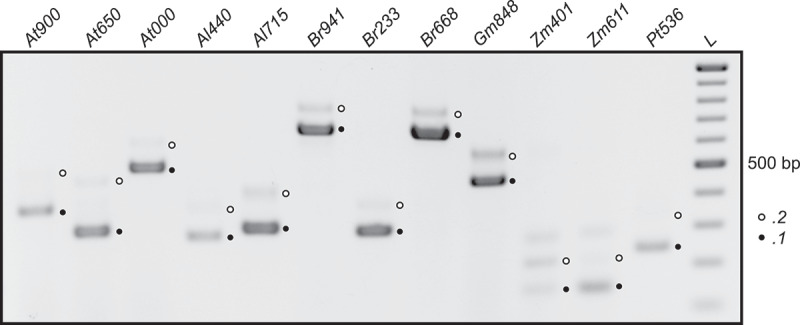
Alternative splicing of *RBP45* homologs in various species. Species were selected to cover different plant lineages and based on availability of genetic material. RT-PCR products were separated on an agarose gel and visualized by ethidium bromide staining. The lower band corresponds to splice variant .*1* (coding in *A. thaliana*) and the upper to variant .*2* (NMD targets in *A. thaliana*) in each lane. Species are: *At900: A. thaliana*, locus tag *AT5G54900; At650: Arabidopsis thaliana, AT1G11650; At000: A. thaliana, AT4G27000; Al440: Arabidopsis lyrata, LOC9300440; Al715: A. lyrata, LOC9328715; Br941: Brassica rapa, LOC103844941; Br233: B. rapa, LOC103843233; Br668: B. rapa, LOC103861668; Gm848: Glycine max, LOC100796848; Zm401: Zea mays, LOC100274401; Zm611: Z. mays, LOC100191611; Pt536: Populus trichocarpa, LOC7458536*. For some samples, the upper band is barely visible, but all bands corresponding to splice variant .*2* were confirmed through sequencing and marked with a white dot. L, size marker in 100 bp increments from 100 to 1000 bp. Gene models for all transcripts are depicted in Supplementary Figure 2 in Supplementary File 1 and sequences are in Supplementary File 3.

Given the predominant conservation of the splice sites in sequences of homologous genes, we investigated AS in the motif-containing transcript region in additional plant species (Figure 4). All tested pre-mRNAs undergo AS, with one protein coding isoform and one that introduces a PTC (Supplementary Figure S2 in Supplementary File S1). As with the *DEAD* motif, this result suggests that AS in the region of the motif is conserved and important.

We hypothesize that the motif might affect splice site selection and therefore affect expression of the gene. We propose that this is done in an auto-regulatory manner, as the parent gene of the motif encodes an RNA-binding protein. Binding of this protein to the RNA motif might be mediated through the predicted secondary structure, and/or lead to an altered structure. This binding could then make the alternative splice sites available or recruit splicing factors and allow inclusion of the CE with its PTC. We are evaluating this hypothesis in a follow-up study (Reinhardt et al., in preparation).

### GRP7&8 motif

A promising secondary structure was predicted for an intronic region inside glycine-rich RNA-binding protein (GRP) genes. Sequences of this motif were found in 91 species of the *Pentapetalae*, the largest eudicot subgroup. Homologues in *A. thaliana* are found in the *GRP7* gene (*AT2G21660*, also called *CCR2*) and its paralog *GRP8* (*AT4G39260*, *CCR1*). *GRP7* and *GRP8* expression follow circadian oscillation [56] and are known to be auto- and cross-regulating in *A. thaliana* [57,58]. An increase in GRP7 or GRP8 protein concentration leads to increased binding of their own transcript, which leads to usage of the alternative 5’ splice site and NMD of the transcript. They are also involved in regulating flowering time [57,59].

**Figure 5.**
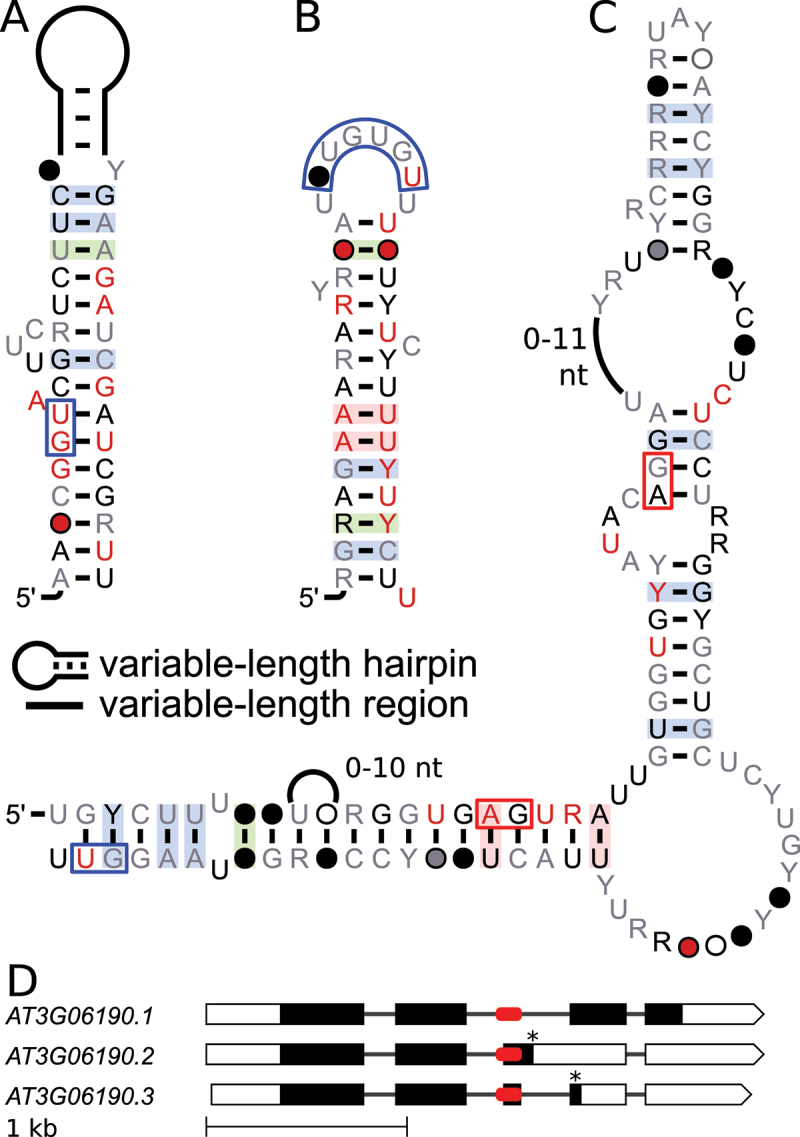
Consensus diagrams of motifs containing alternative splice sites that are involved with NMD. A: *GRP7&8* motif; B: *PTB2* motif; C: *BPM1&2* motif. Annotations are the same as in Figure 1. Splice sites are highlighted; red box: 3’ splice site, blue box: 5’ splice site. D: Gene model for *A. thaliana BMP2* (*AT3G06190*), asterisks indicate positions of in-frame PTCs. Rounded red rectangle indicates the motif; other details of gene model structure described in Figure 1.

A 20-base-pair-long stem showing signs of covariation was predicted as secondary structure for this motif (Figure 5(a)), although R-scape evaluates this stem’s covariation to be barely significant at a 0.05 threshold (E-value: 3.6 × 10^−2^). An alternative 5’ splice site near the 5’ end of the stem is annotated in some sequences, and the relevant ‘GU’ dinucleotide is ubiquitously conserved (Figure 5(a), blue box). This is consistent with the hypothesis that this splice site is used in all sequences as an alternative splice site. In *A. thaliana*, this ‘GU’ dinucleotide is annotated as a cryptic alternative 5’ splice site, i.e. its usage leads to degradation of the transcript through the NMD pathway [57]. Given the previously mentioned conservation, this function might also be conserved in all other sequences and usage of the splice site might be dependent on the motif.

Staiger et al. [58] have shown that a stretch of 23 nucleotides, 106 nucleotides downstream of the alternative 5’ splice site, is part of the binding sites for GRP7 and GRP8 proteins. This area is located 61 nucleotides downstream of the motif. Given the structural prediction and the binding sites of GRP7 and GRP8, a possible regulatory mechanism of this motif is that its secondary structure occludes the alternative splice site and prevents its usage. In the case of abundant GRP7 or GRP8, binding downstream of the motif might destabilize the predicted structure, freeing the splice site for recognition and usage.

### PTB2 motif

Another promising candidate was found in introns of polypyrimidine tract binding protein (PTB) genes in 93 *Pentapetalae* species. In *A. thaliana*, the motif is found in *PTB2* (*AT5G53180*) and it overlaps the end of a CE. All genes in which this motif is found are annotated as ‘polypyrimidine tract binding protein homolog 2’, signifying that they are all homologs of *AT5G53180*. We did not find representatives of this motif in annotated *PTB1* or *PTB3* homologs. The *PTB2* gene is auto- and cross-regulated by its protein and *PTB1*’s protein and the CE is included upon PTB1/2 regulation, leading to NMD [45,60,61]. Rühl et al. also showed that PTB proteins bind to stretches of pyrimidines around the CE of *A. thaliana PIF6*. It was recently demonstrated that the PTB-dependent inclusion or skipping of a CE in *A. thaliana* depends on the binding position of the PTB protein [62].

The secondary structure is predicted to be a 14-base-pair-long hairpin loop, with the 5’ splice site of the CE located in the terminal loop (Figure 5(b), blue boxes). Most sequences conserve a ‘GUGUGU’ consensus in the terminal loop providing three potential splice sites. As with the *45ABC* motif, these may all be part of the same splice site, with only the first ‘GU’ being used for splicing. Some sequences contain fewer ‘GU’ dinucleotides in the terminal loop, but always preserve at least the one on the 3’ end of the terminal loop. While the original prediction was qualitatively compelling, additional homology searches did not result in a convincing structure (E-value: 0.87). However, given the position of the motif around an alternative splice site that is involved with NMD, we found the motif nonetheless of potential interest. The 3’ part of the stem also contains a long, conserved stretch of pyrimidines that can serve as a PTB1/2 binding site [62], which, in turn, can affect splicing. The *PTB2* motif might thus play a role in regulating PTB2 expression by affecting binding of PTB1/2 to the mRNA.

### BPM1&2 motif

We found a promising motif in the second intron of BTB-POZ and MATH (BPM) domain protein genes 1 and 2 (*BPM1*&*2*) in 31 species from the rosids clade, a large subgroup of the *Pentapetalae*. BPM proteins form part of Cullin E3 ubiquitin ligase complexes and bind at least three families of transcription factors: APETALA2/Ethylene Responsive Factor (AP2/ERF) class I, homeobox-leucine zipper and R2R3 MYB [63]. Škiljaica et al. [63] showed that the binding of BPM proteins to these transcription factors plays an important role in plant flowering, seed development, and abiotic stress response.

In roughly half of the sequences, the alternative 3’ and 5’ splice sites of a cassette exon are annotated in RefSeq, with four of those having an additional alternative 3’ splice site annotated (Figure 5(c), red and blue boxes). In *A. thaliana*, the cassette exon is involved in NMD in *BPM1* (*AT5G19000*), but involvement of NMD in *BPM2* (*AT3G06190*) is unclear [45]. However, two transcript variants with PTCs were identified in RefSeq for *AT3G06190* in which the CE region is included (Figure 5(d)).

The secondary structure is predicted as a single hairpin loop, broken up by one small and two large bulge loops. While the original prediction was compelling and the location of the motif was interesting due to the nearby alternative splice sites, none of these stems produce significant E-values (E-value: 0.23). We hypothesize that this secondary structure influences inclusion of the CE and therefore affects expression of the *BPM1* and *2* genes.

### StrucRNAs not associated with NMD

**Figure 6.**
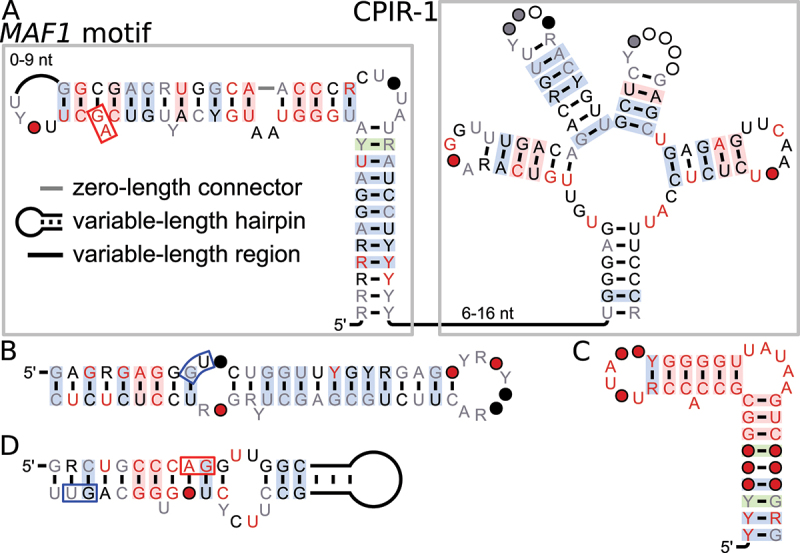
Consensus diagrams of motifs found in consistent locations in homologous genes, without information on alternative splicing. A: *MAF1* motif and the nearby *CPIR-1* found by a previous study [13]; B: *UbiE2* motif; C: *Kre33* motif, D: *STK38* motif. Annotations are the same as in Figure 1. Splice sites are highlighted; red box: 3’ splice site, blue box: 5’ splice site.

The other five strucRNA candidates that are found in consistent locations of homologous or functionally related genes are not nearby NMD-associated alternative splice sites annotated in *A. thaliana* by Drechsel et al. [45] or are not present in *A. thaliana*. While the locational homology can serve as an indicator of *cis*-regulatory RNAs, we also found snoRNAs, which act in *trans*, in consistent locations in homologous genes. In the absence of NMD association and other features of a *cis*-regulatory function, we refrain from classifying strucRNAs as *cis*-regulatory. They might instead act in *trans*, being released when their parent genes are transcribed and the resulting pre-mRNAs are processed. These motifs’ secondary structure predictions are shown in Figure 6. Curiously, all of the secondary structures are predicted as a hairpin loop with a bulge loop in the centre. All but the *STK38* motif contain significant structures according to our R-scape-based process.

**The *MAF1* motif** was found in introns of *MAF1* homolog genes in most species from the *Magnoliophyta* clade (105). These genes serve as repressors of RNA polymerase III transcripts. Disruption of the *MAF1* gene or RNAi-mediated depletion of its transcript is well tolerated and confers a modest growth advantage without compromising resistance to common biotic and abiotic challenges [64].

The area near the terminal-loop of the predicted structure conserves an ‘AG’ in all sequences in this motif (Figure 6(a), red box). In *Hevea brasiliensis* (rubber tree), but not in other species, this ‘AG’ is annotated as an alternative 3’ splice site in RefSeq. When we extended the sequences and used CMfinder to predict secondary structures, we found an additional secondary structure prediction about 10 nucleotides downstream of the *MAF1* motif. This structure was predicted earlier as a putative tRNA-like ncRNA and named *CPIR-1* [13] (RNAcentral ID: URS0000C5172F_2711).

**The *UbiE2* motif** is found in the 5’ UTR of 80 *UEV1D–4* ubiquitin E2 genes in species from the *Pentapetalae* clade. These genes promote lysine-63-linked polyubiquitination and are involved in the DNA damage response [65]. Splice site annotations from RefSeq show an alternative 5’ splice site in *Ricinus communis* and *Olea europaea*, two species from the Eudicots, that is conserved in the motif (Figure 6(b), blue box). The conservation in the lower part of the stem is strongest on the 5’ side of the motif, where the highly conserved ‘GU’ dinucleotide of the potential splice site is located.

**The *Kre33* motif** (Figure 6(c)) was found in three species from the *Mamiellales*, a group of green algae; *Micromonas pusilla*, *Micromonas commoda* and *Bathycoccus prasinos*. The sequences are all in introns of similar genes, based on protein BLAST (data not shown), with only the gene in *Bathycoccus prasinos* having a function annotated in RefSeq. That gene is annotated as nucleolar ATPase *Kre33*, which is predicted in OrthoDB [66] to be an ortholog of GNAT acetyltransferase.

**The *STK38* motif** was found in the 3’ UTRs of AGC-serine/threonine-protein kinase 38like (*STK38*) genes in 111 out of 113 species from the *Magnoliophyta* clade. These kinases are essential for many uni- and multicellular organisms [67]. The ‘AG’ dinucleotide at the 5’ end of the motif is conserved in all sequences (Figure 6(d), red box) and annotated as an alternative splice site in *Musa acuminata*, a species of banana. A ‘GU’ dinucleotide at the 3’ end of the motif is conserved in about 90% of sequences (Figure 6(d), blue box) and annotated as a constitutive 5’ splice site in *Populus alba* and *Olea europaea*, two trees from the Eudicots. It therefore seems possible that the motif contains a CE whose inclusion is dependent on the motif’s secondary structure, but no additional evidence to support this hypothesis was available. While the motif was originally compelling, additional sequences from homology searches did not support the predicted secondary structure with covariation, as mirrored by the evaluation from R-scape (E-value: 0.54).

**Figure 7.**
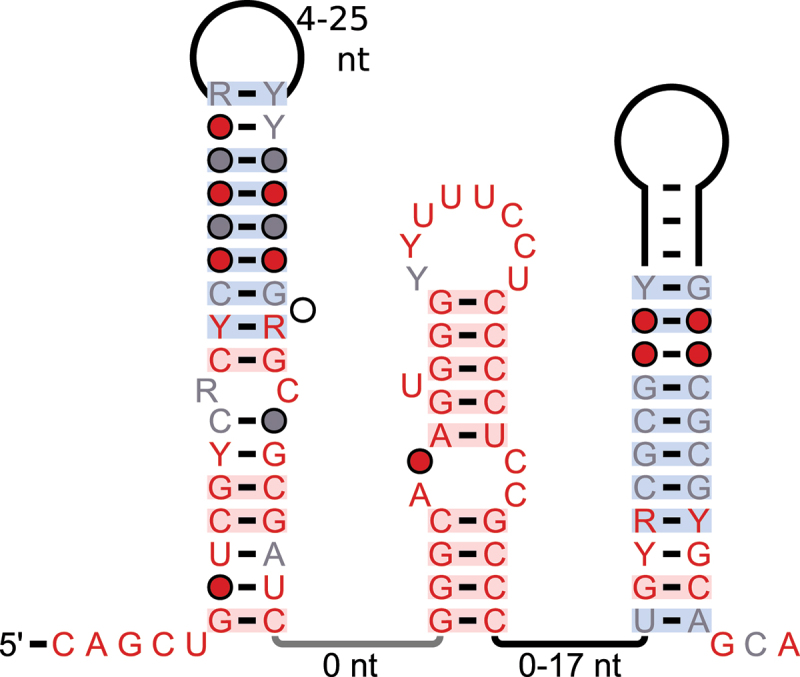
Consensus diagrams of the *MicMet* motif. Annotations are the same as in Figure 1 and 6.

**The *MicMet* motif** was identified in the 3’ UTR of two different genes: an *S-*adenosylmethionine (SAM) synthetase in *M. commoda* (Locustag: *MICPUN_109618*) and a cystathionine gamma-synthase in *M. pusilla* (*MICPUCDRAFT_25455*). The structure features three conserved hairpins, with the middle one showing the most sequence conservation (Figure 7). The motif appears twice in *M. pusilla*, 20 nucleotides apart. Although we found only three examples in RefSeq, BLAST searches of *Chlorophyta* transcriptome shotgun assembly (TSA) datasets using the NCBI web site and Infernal searches of a metagenomic dataset from a previous study [52] detected several similar sequences.

The two genes associated with the *MicMet* motif encode distinct enzymes involved in methionine biosynthesis, which suggests that the motif might play a role in regulation of this pathway. Indeed, genes encoding these enzymes are frequently regulated by bacterial riboswitches that bind SAM or the related metabolite *S*-adenosylhomocysteine (SAH) [68]. However, experimental analysis of *MicMet* motif RNAs did not reveal evidence for binding of SAM or SAH (data not shown). Therefore, we hypothesize that the *MicMet* motif regulates genes using another mechanism, e.g. via a SAM-binding protein.

### Putative snoRNAs

We found five motifs whose features suggest that they are snoRNAs, i.e. they contain conserved sequence motifs specific to C/D box snoRNAs or H/ACA box snoRNAs and show similar conserved structural features [69]. According to our R-scape-based method, all secondary structures predicted for these putative snoRNAs are statistically significant with E-values less than 2 × 10^−3^. All of these candidates are found in similar locations of homologous genes, which is in line with our method of finding *cis*-regulatory strucRNAs and it has been shown that snoRNAs can even affect expression of their host gene through AS and NMD [70,71]. However, in their primary function, snoRNAs operate in *trans*, and a *cis* function would need to be validated experimentally.

**Figure 8.**
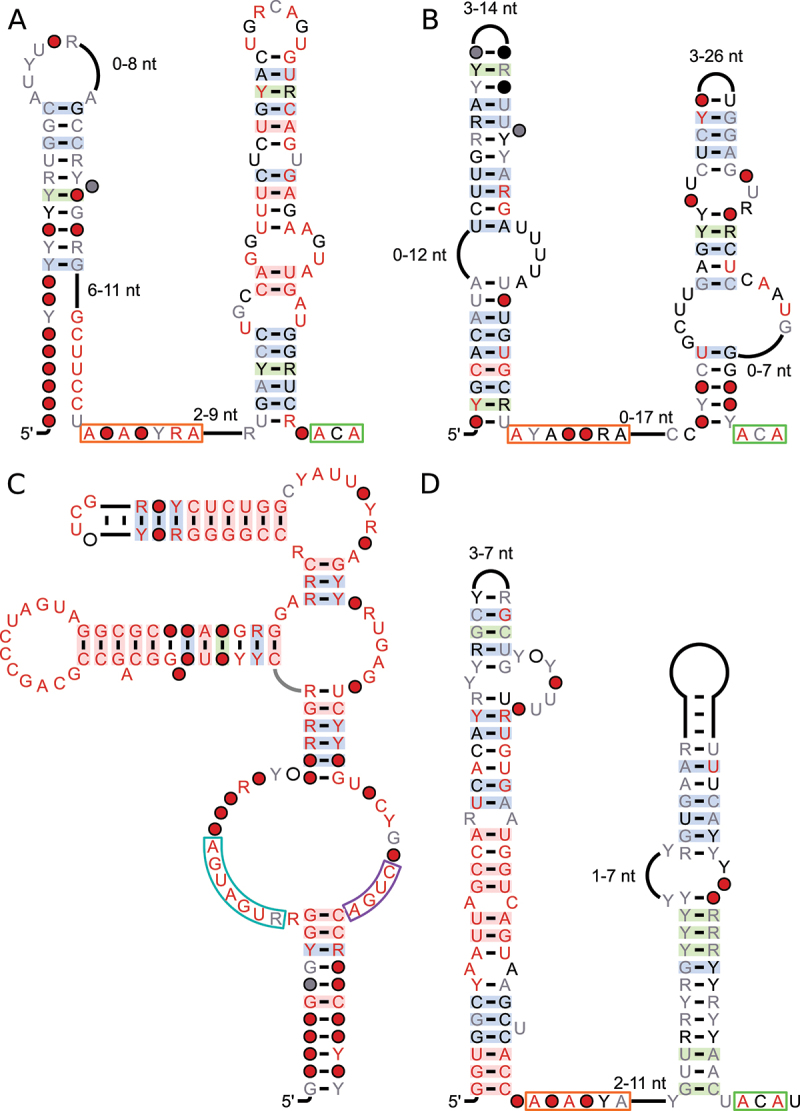
Consensus diagrams of snoRNA candidates. A: *PRP38-HACA* motif; B: *MPCRibo-HACA* motif; C: *Mamiellales-CD* motif, D: *EIF1&2-HACA* motif. Annotations are the same as in Figures 1 and 6. Sequence motifs typical for snoRNAs are highlighted: orange: H-box, green: ACA-box, turquoise: C-box, purple: D-box.

**The *PRP38-HACA* motif** was found in the last intron of pre-mRNA-splicing factor 38 (*PRP38*) family protein genes from 102 species from the *Magnoliophyta* clade. PRP38 is required for pre-mRNA splicing and maintenance of stable U6 small nuclear RNA levels [72]. The motif contains both an H-box and an ACA-box (Figure 8(a)). These are separated by a stem supported by covariation. This stem also contains a conserved bulge loop, further implying the presence of an H/ACA-box snoRNA. However, the stem predicted upstream of the H-box is poorly supported. The predicted secondary structure still bears resemblance to a H/ACA snoRNA. The H box’s consensus is ‘ANANNRA’, which is not typical for H-boxes. However, it was shown that ‘ANANA’ and ‘ANANNNA’ can both serve as valid H-boxes [73], so the *PRP38-HACA* motif’s consensus could be valid. Similarly, the ‘C’ from the ACA-box is also not completely conserved, but it was shown that alternative sequences (‘AUA’, ‘AAA’, or ‘AGA’) can be found [74]. Both of these differences in the boxes also apply to the *MPCRibo-HACA* motif and *EIF3G1*&*2-HACA* motif.

**The *MPCRibo-HACA* motif** is predominantly found in three different genes, encoding mitochondrial phosphate carrier protein 3, 40S ribosomal proteins and 60S ribosomal proteins. One of the main targets for modification by snoRNAs are ribosomal RNAs (rRNAs) [75]. It is therefore of interest that this snoRNA candidate is part of ribosomal protein genes whose product forms a complex with rRNAs, and such a correlation has been observed before [76,77]. The predicted secondary structure matches the features of an H/ACA-box snoRNA (Figure 8(b)).

Hits to this motif can be found in 49 species of the *Magnoliophyta* clade. The motif is always in a similar, intronic, region in each of these genes: sequences in mitochondrial phosphate carrier protein 3 and 40S ribosomal proteins are always in the third-to-last intron, while sequences in 60S ribosomal proteins are in the last intron. All of these introns are much larger than other introns in the same genes. Some sequences from this motif are found in proximity to known snoRNA families RF00359 and snoU30, both of which are C/D-box snoRNAs.

**The *Mamiellales-CD* motif** was found in three species from the *Mamiellales* (an order of green algae); *M. pusilla*, *M. commoda* and *B. prasinos*. The sequences were not found in consistent positions relative to homologous genes. The motif exhibits the standard C/D-box snoRNA structure: an internal loop containing a C-box and D-box, brought together by a surrounding stem (Figure 8(c)).

**The *EIF3G1*&*2-HACA* motif** was found in the first intron of the two genes that code for the G subunit of eukaryotic initiation factor 3 (*EIF3G1* and *EIF3G2*) from 68 species of the *Magnoliophyta*. The motif conforms to the typical structure of a H/ACAbox snoRNA. The sequence in *A. thaliana* (*AT5G01135*) is annotated as a snoRNA in RNAcentral (RNAcentral ID: URS0000A76B6C_3702) [46]. We expand upon previous results by predicting a secondary structure and finding homologs in 67 other species (Figure 8(d)).

### Trans strucRNAs

Our search strategy was directed at finding strucRNAs that are in *cis* to homologous genes. However, we found two strucRNA candidates (Fig.) that are not consistent with this property. We therefore do not associate them with AS or other forms of *cis*-regulation. Both predicted secondary structures are significant based on R-scape’s evaluation.

**Figure 9.**
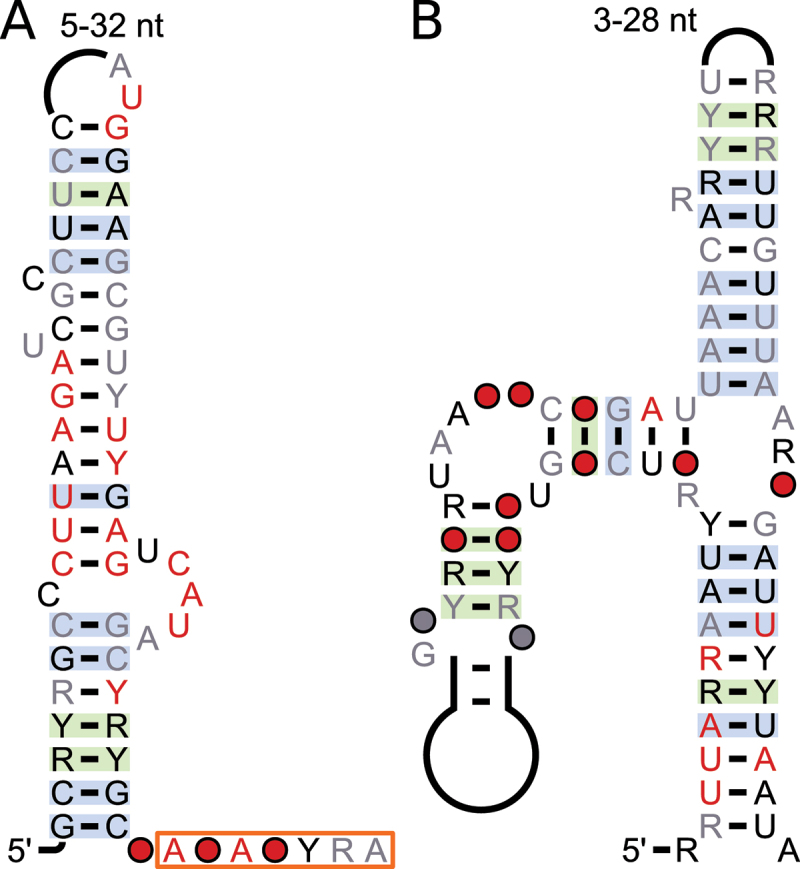
Consensus diagrams of strucRNAs that are not in consistent locations around homologous genes. A: *Viridi-trans* motif; the potential H-box is highlighted in orange. B: *Brassica-trans* motif. Annotations are the same as in Figures 1 and 6.

**The *Viridi-trans* motif** was found in 119 species from the *Viridiplantae* clade, but not in a consistent gene context. The motif is predicted as a long hairpin, divided by a large bulge-loop (Figure 9(a)). This bulge-loop contains very conserved sequence elements. Downstream of this stem, a sequence similar to the H-box of the previously presented H/ACA-box snoRNA candidates can be found, with half the sequences conserving the ‘ANANNA’ consensus, while the other half shows a ‘ANANNNA’ consensus (Figure 9(a), orange box, also see *PRP38HACA* motif). Both of these features are indicative of a H/ACA-box snoRNA, however we were unable to find two additional components of H/ACA-box snoRNAs: a convincing stem downstream of the H-box and a conserved ACA-box. These findings make it unlikely to function as an H/ACA-box snoRNA. However, many of the sequences of this motif are positioned close to known snoRNAs, namely snoR21 and snoZ266, both of which are C/D-box snoRNAs.

**The *Brassica-trans* motif** (Figure 9(b)) is only found in members of the *Brassicaceae* family, e.g. *A. thaliana*, for a total of 9 different species. The sequences do not appear in a consistent context, making a *cis*-regulatory function unlikely. Instead, there are many copies of the motif in each species of the *Brassicaceae*.

## Discussion

### Findings

By using a comparative genomics-based approach on the 130 plant genomes available in RefSeq, we identified 16 structured regions of non-coding RNA that we hypothesize to be functional. Five of those are likely to function in *cis* by regulating the expression of their parent genes through AS. Five other motifs were found in consistent locations of homologous or functionally related genes, but no alternative splice sites were annotated in their vicinity, making evidence for a *cis-*regulatory function ambiguous. We also predict four snoRNAs, three of which are completely novel, while the fourth was previously known in far fewer species. In contrast, the remaining two strucRNA candidates that we present were not found in a consistent genomic context, suggesting a *trans* function.

In addition to these novel predictions, many of the top-scoring motifs contained sequences that overlap different kinds of already known RNAs. These include various miRNAs, tRNAs and snoRNAs. We also found motifs corresponding to the previously discovered *P5SM* [9] and TPP riboswitch [5–7].

Compared to previous studies in bacteria, this is a very small number of predictions for strucRNAs. For example, Weinberg et al. [25] previously predicted 224 strucRNAs in bacteria, with roughly twice as much non-coding sequence as input. In comparison to that study, we also found that covariation is less pronounced in plants, especially for the putative *cis*regulatory strucRNAs. Our analysis with R-scape produced many insignificant aggregated E-values for those motifs, using a threshold of 0.05. The snoRNA candidates, however, show significant E-values of less than 2 × 10^−3^. Applying R-scape without aggregation and while considering all base pairs to the motifs from the previously mentioned study by Eckert and Weinberg [52], revealed an average of 6.7 significantly covarying base pairs for their 23 motifs from bacteria, while the 16 motifs that we presented here only have 0.625 on average. These observations confirm once again that covariance is useful for the discovery of strucRNAs, even if its extent might be limited in plants, especially for *cis-*regulatory strucRNAs, and are in line with previous observations on the TPP riboswitch and the *P5SM*.

### Limits

There are limitations to our method that likely affected the number of promising candidates that we found. The first limitation is the availability of data. The RefSeq version that we used (version 204) contains 130 plant genomes, while there are more than 10,000 fully annotated bacterial genomes available in the same version of RefSeq [34]. With more species available, covariation might be more apparent and make previously less supported motifs more visible. To reduce the false-positive rate, structural predictions on only one or two sequences were immediately discarded. Availability of sequences from more species or subspecies would presumably result in more clusters passing the threshold of three sequences. For example, we did not find a compelling helix structure in the 5’ UTR of the key heat shock regulator *HsfA2* in *S. lycopersicum* that could correspond to the previously identified thermosensor [8]. If this RNA structure is only present in *S. lycopersicum* subspecies, then it would not pass the 3 species threshold that we employed, since only one *S. lycopersicum* subspecies is fully annotated in our dataset.

A related limit is the relatively low evolutionary diversity of plants compared to bacteria, resulting in fewer mutations that could strengthen a covariation signal, e.g. bacteria evolved around 3 billion years ago [78], while plants only evolved 1 billion years ago [79]. Additionally, there is less time for subgroups of plants to develop novel strucRNAs. This situation could likely be somewhat improved with additional sequences in subgroups such as algae or ferns that are currently unavailable or under-represented. However, most plant clades are already represented in RefSeq. These factors may severely impact the effectiveness of comparative genomics in plants, since a covariation-based comparative genomics strategy relies on the availability of usefully diverged sequences to be able to identify conserved patterns.

Another limitation is that we generated the input clusters of genes through protein similarities from *A. thaliana* and *O. sativa*. While these parameters were chosen carefully to both maximize potential candidates and reduce false-positives, this limits the motifs we can predict to all classes of proteins that are annotated in either species. For example, any genes unique to the eudicotyledons, but absent from *A. thaliana* would automatically be missing from our search. If our method was modified such that strucRNAs from such groups could be found, these predictions would, however, be hard to evaluate due to the small number of existing homologs and their limited divergence. We did run a version of the pipeline on green algae (*Chlorophyta*), and found a modest number of motifs with significant covariation. This covariation was greater when additional homology searches also revealed sequences in other plant species. Our main, protein-oriented method however also predicts motifs with sequences from the *Chlorophyta*, as long as there are homologs in *A. thaliana* or *O. sativa*.

The biggest limit on our results might thus be its focus on covariation. This factor could explain the lack of motif predictions overlapping some previously known strucRNAs and there could be as-yet undiscovered strucRNAs in plants that we failed to detect because they did not exhibit covariation.

Additionally, our analysis and evaluation of predictions was aided by AS data in *A. thaliana* [45] and availability of such auxiliary data for more plant species would allow for a broader automation and annotation of predictions. This can help to reduce false-positives or prioritize motifs with a clearer hypothesis about their function and would therefore allow for less-strict initial criteria.

### Prospects

Especially given the lack of known structural motifs in plants that affect their transcript in *cis*, it would be interesting to estimate how many more strucRNAs remain to be discovered. The small number of candidates predicted by our work and previous studies limits our knowledge on *cis*-regulatory strucRNAs in plants. The modest amount of covariation observed raises the possibility of many strucRNAs that did not exhibit enough covariation to be discovered. Therefore, it remains an open question how many unknown strucRNAs remain in plants. With some of these strucRNAs clearly demonstrating the importance of pre-mRNA secondary structures in regulating gene expression, it is likely that more strucRNAs in plants remain to be discovered. However, such discoveries will likely require alternate bioinformatics and/or experimental methods capable of reliably detecting strucRNAs with low false-positive rates. The availability of more genomic and transcriptomic data, as well as more information about plant strucRNAs, would also aid in the detection.

Despite the limitations of covariation in plants, described above, the 16 motifs presented in the present study can serve as starting points for more detailed experimental analyses of their effects. Two motifs (*DEAD*, *45ABC*) are already being studied in more detail (Burgardt et al. in preparation, Reinhardt et al. in preparation), and other predictions are available for follow-on work. Among the probably non-*cis*-regulatory RNAs, the snoRNAs exhibit significant covariation, and are the strongest candidates for experimental investigation.

## Supplementary Material

Supplemental Material

## Data Availability

The authors confirm that the data supporting the findings of this study are available within the article and its supplementary materials. All supplementary materials are described in more detail in Supplementary File S1. Gene models for AS analyses are available in Supplementary File S1. Transcript sequences for AS analyses are available in Supplementary Files S2 and S3. Alignments in machine-readable format (Stockholm) in Supplementary File S4. Most sequence accessions in alignments come from RefSeq, but we used metagenomic sequences for some motifs present in algae to increase covariation. Those sequences are available from the NCBI. Figures of motif secondary structures in Supplementary File S5. Additional information and notes about the motifs are in Supplementary Table S1. Primers used for PCR are in Supplementary Table S2.
