# Supplementary material for: Identification and characterization of new structured RNA classes in plants
Source: RNA Biol. 2025 Jun 30;22(1):1–16. doi: 10.1080/15476286.2025.2523696 (PMC12218528; doi:10.1080/15476286.2025.2523696)
Supplement: Supplemental Material [file KRNB_A_2523696_SM4654.zip › Supplementary_File_1.pdf]

Supplementary materials for
Identification and characterisation of new structured RNA classes in plants

This file describes the supplementary materials for the paper.

- **Supplementary File 1**: (Current file) contains Supplementary Figures, describes other Supplementary Files, provides additional information for the main text; PDF format (.pdf)
- **Supplementary File 2**: mRNA sequences for verified transcript variants of DRH1 homologs; in fasta format (.fasta)
- **Supplementary File 3**: mRNA sequences for verified transcript variants of RBP45A/B/C homologs; in fasta format (.fasta)
- **Supplementary File 4**: Zip-compressed folder containing sequence alignments of the presented motifs; in Stockholm alignment format (.sto)
- **Supplementary File 5**: Zip-compressed folder containing graphics of secondary structure predictions of the presented motifs; in SVG format (.svg)
- **Supplementary Table 1**: Table containing additional information on the presented motifs; Microsoft Excel Worksheet (.xlsx)
- **Supplementary Table 2:** Table containing the primers used for co-amplification PCR for alternative splicing analysis; tab-separated plain text file (.tab)

Supplementary File 1

Alternative splicing analysis

Using RT-PCR and sequencing, we analysed splicing patterns of genes containing the *DEAD* motif (Fig. 2) and the *45ABC* motif (Fig. 4). We provide the corresponding gene models in Sup. Fig. 1 and Sup. Fig. 2, for genes containing the *DEAD* motif and genes containing the *45ABC* motif, respectively. Additionally, we provide the mRNA sequences of the transcript variants that could be verified in Supplementary File 2 and Supplementary File 3 in fasta format.

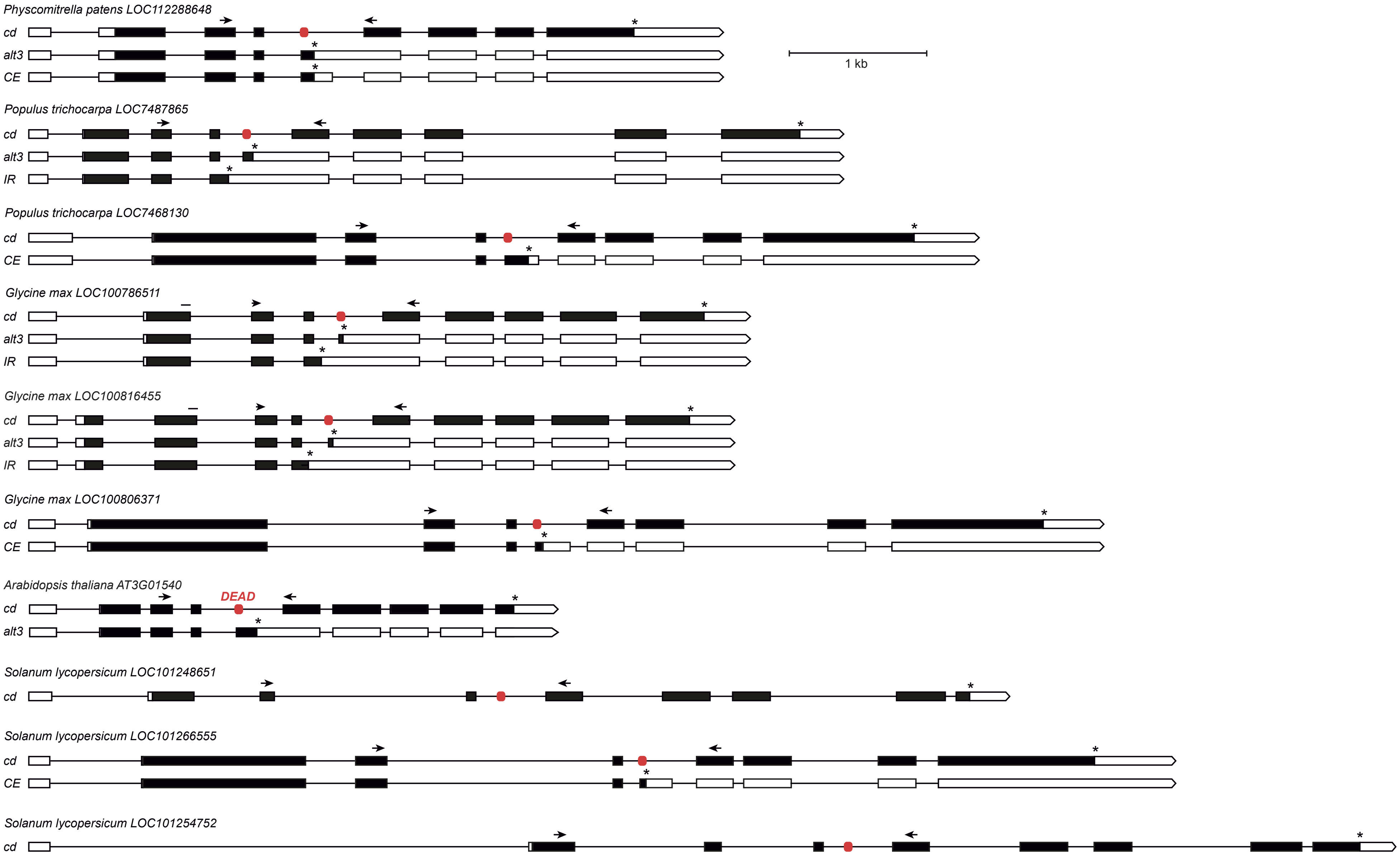

Supplementary Figure 1: Alternative splicing in *DEAD* motif-containing regions of the DRH1 homologs analysed in Figure 2. Gene models of splicing variants *cd* and *alt3* with exons (boxes) and introns (lines) are shown; some genes also have intron retention (*IR*) or cassette exon (*CE*) variants. Boxes

correspond to UTRs (white) and coding sequences (black). Rounded red rectangles indicate the motif sequence in each gene. Asterisks indicate positions of translational stop codons, arrowheads show binding sites of primers for co-amplification PCRs. Sequences of all transcripts are in Supplementary File 2.

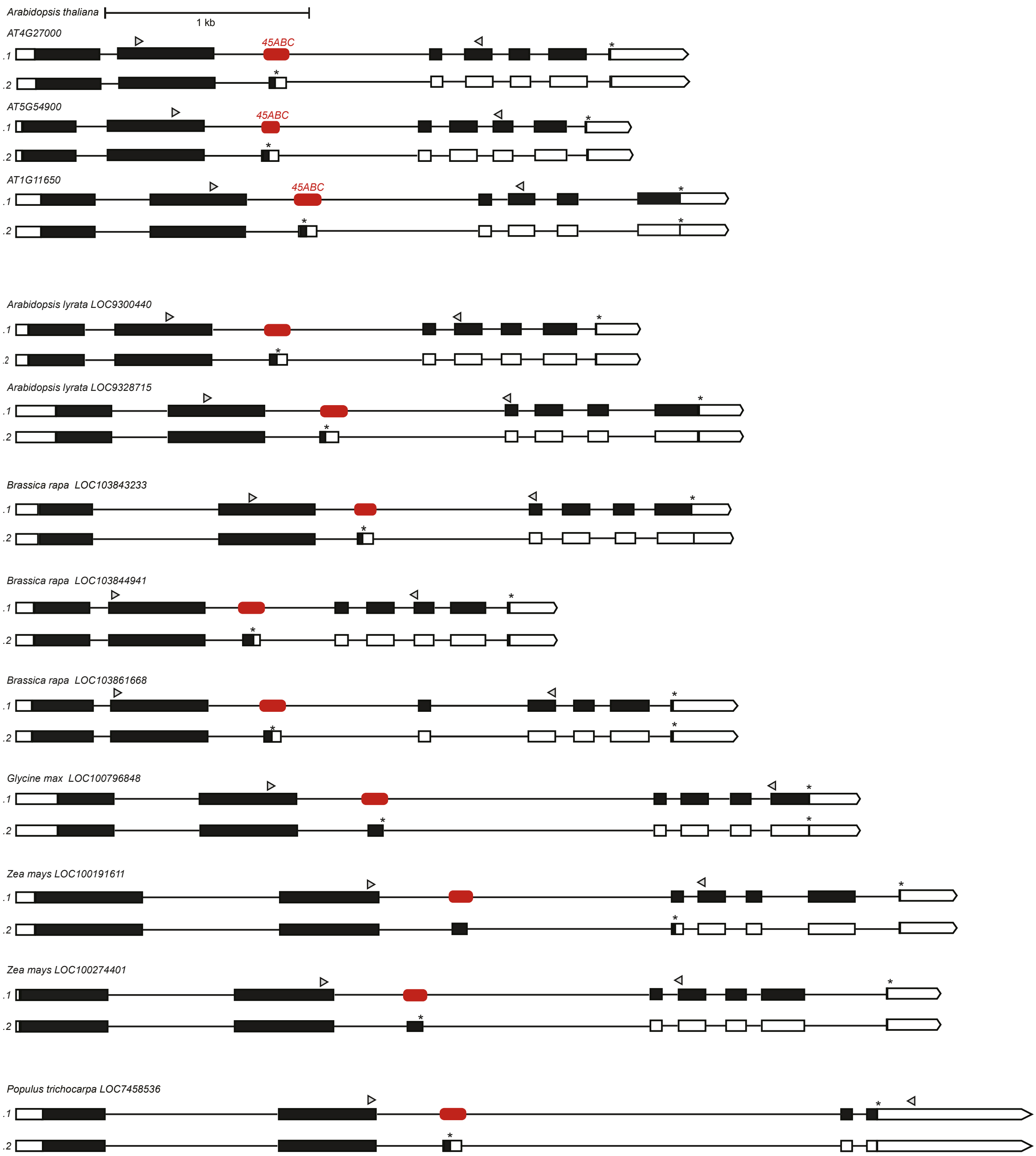

Supplementary Figure 2: Description as Figure 1. Alternative splicing in *45ABC* motif-containing regions of the RBP45A,B/C homologs analysed in Figure 4. Gene models of splicing variants *.1* and *.2* with exons (boxes) and introns (lines). Boxes correspond to UTRs (white) and coding sequences (black). Rounded red rectangles indicate the motif sequence in each gene. Asterisks indicate positions of translational stop codons, arrowheads show binding sites of primers for co-amplification PCRs. Sequences of all transcripts are in Supplementary File 3.

Description of Supplementary File 4:

We provide sequence alignments in Stockholm format for all presented motifs in Supplementary File 4. The Stockholm alignment format is a plain text format, and as such readable by any text editor with a fixed-width font, but should be opened with a capable alignment editor, such as RALEE. It provides aligned sequences and a consensus secondary structure for said alignment.

These alignments contain all sequences that passed an E-value threshold of 0.05, based on the covariance model for the alignment.
This resulted in the inclusion of sequences that in our opinion do not fit the motif's pattern well, but removing such sequences based on such a subjective criterium would have biased our results.

Additional note on the *DEAD* motif:
Due to nucleotide conservation in the stem of the *DEAD* motif, some sequences are shown with a terminal loop shorter than 3 nucleotides. This is biologically not possible and only displayed this way due to the sequence conservation in the stem.

Description of Supplementary File 5:

We provide graphical depictions of the predicted secondary structures for all motifs in SVG format in Supplementary File 5. These figures were created with R2R (Weinberg & Breaker, 2011) and annotations were added or improved with Inkscape (https://inkscape.org/).

The figures use colouring to show various features of the alignment:
Colouration of the nucleotides shows their conservation, with red indicating at least 97% of nucleotides at this position are the same, black 90% and gray 75%.
Basepairs are shaded based on the observed covariation, where green indicates covariation based on R-Scape's evolutionary method (Gao et al., 2021), blue indicates covariation based on R2R's simple method and red indicates that no mutations are observed in this basepair.
Red, black and grey filled circles indicate the presences of nucleotides if they are not conserved at 97%, 90% and 75%, respectively.

Splice site marker dinucleotides have been highlighted if they were found near the motif:
Red boxes indicate the "AG" dinucleotide in front of 3′ splice sites and blue boxes indicate the "GU" dinucleotide after a 5′ splice site.
For snoRNAs, the conserved boxes are highlighted: H-box: Orange, ACA-box: Green, C-box: Turquoise, D-box: Purple.
